# Personalized Type 1 Diabetes Management: Reinforcement Learning–Based Insulin Dosing and Glucose Forecasting

**DOI:** 10.2196/79195

**Published:** 2026-06-03

**Authors:** Ernest M Taku, Vibhuti Gupta, Ashutosh Singhal

**Affiliations:** 1 Department of Biomedical Data Science, School of Applied Computational Sciences, Meharry Medical College, Nashville, TN, United States; 2 Department of Biostatistics & Data Science, School of Public and Population Health, The University of Texas Medical Branch at Galveston, 301 University Boulevard, Galveston, TX, 77555-1150, United States, 1 8065006843

**Keywords:** personalized insulin dosing, reinforcement learning, deep Q-network, adaptive insulin regimens, machine learning, health care, artificial intelligence

## Abstract

**Background:**

Optimizing insulin dosing and predicting future glucose levels for people with type 1 diabetes is challenging due to the dynamic nature of glucose metabolism. Traditional static insulin regimens fail to adapt to individual variability in diet, physical activity, stress, and metabolic fluctuations, leading to suboptimal glycemic control. Reinforcement learning (RL) offers a promising alternative by enabling personalized, real-time insulin adjustments that improve the balance between hyperglycemia and hypoglycemia.

**Objective:**

This study aims to develop a deep Q-network (DQN)–based RL system that dynamically personalizes insulin dosing recommendations using continuous glucose monitoring data, meal intake, and physical activity levels. By leveraging real-time data, the model adapts to patients’ evolving physiological states, enhancing glucose control and patient safety.

**Methods:**

We used the OhioT1DM dataset (2018 and 2020), which includes 8 weeks of continuous glucose measurements, insulin dosing records, and physical activity data for twelve people with type 1 diabetes. The RL agent was designed with a state representation consisting of recent blood glucose levels, insulin doses, and lifestyle factors over a 2-hour window. The 2-hour window was selected based on the known pharmacodynamic profile of rapid-acting insulin (peak action within 90‐120 min), as well as the typical lag in glycemic response following meals or exercise. This window size captures both recent and delayed physiological effects while balancing data density and model stability. The action space included discrete insulin dose recommendations (eg, 0.5 U, 1 U, and 1.5 U). A reward function incentivized glucose levels within the target range (70‐180 mg/dL) while penalizing extreme deviations. The DQN model was trained to maximize reward by learning optimal dosing strategies through iterative trial and error.

**Results:**

Performance evaluation was conducted using both qualitative and quantitative metrics. Time-series analysis compared actual and predicted glucose levels, demonstrating effective glucose regulation. The RL model achieved a mean glucose level of 80.06 mg/dL, with a reward score of 10 during evaluation, indicating that most glucose predictions were maintained within the desired clinical range. This suggests the model has learned to regulate blood glucose effectively through adaptive insulin dosing. The root mean square error (12.39 mg/dL) was slightly higher than the mean absolute error (9.85 mg/dL), indicating stable predictions. Additionally, the percentage time in target range was 64.06%, suggesting that the model-maintained glucose within the clinically safe range for a majority of the time.

**Conclusions:**

The DQN-based RL model demonstrated its effectiveness in personalized insulin dosing while minimizing the risk of hypo- and hyperglycemia. This suggests the model has learned to regulate blood glucose effectively through adaptive insulin dosing. This approach represents a significant advancement over conventional methods, offering a scalable and adaptive strategy for real-world diabetes management, along with enhancing clinical trust and transparency through explainability techniques.

## Introduction

### Background

According to the National Institute of Diabetes and Digestive and Kidney Diseases, diabetes is a chronic condition affecting more than 38.7 million people in the United States, with approximately 1.6 million diagnosed with type 1 diabetes (T1D) [1]. T1D is an autoimmune condition where the body’s immune system mistakenly attacks and destroys the insulin-producing beta cells in the pancreas, resulting in little to no insulin production in the body [2]. Insufficient insulin levels in individuals with T1D can cause hypoglycemia (ie, low blood sugar), hyperglycemia (ie, high blood sugar), and ketoacidosis (ie, ketone development), with potential impacts on vital organs including the heart, kidneys, eyes, and feet. Although the underlying cause of T1D is still unknown, it is widely accepted that both genetic susceptibility and environmental influences contribute significantly to its development [3]. Management of T1D primarily involves regulating blood glucose (BG) levels through insulin therapy, nutritional adjustments, physical activity, and routine glucose monitoring.

Insulin is a hormone the body uses to allow sugar (glucose) to enter cells to produce energy, and it plays a critical role in T1D. Thus, insulin dosing through injections or an insulin pump is the primary treatment to compensate for the body’s inability to produce insulin. However, managing diabetes effectively involves precise insulin dosing to maintain optimal BG levels [4]. Due to variability in patients’ responses to insulin based on factors such as diet, physical activity, stress, and metabolic fluctuations, the challenge of personalizing insulin dosing is critical for improved patient outcomes. Despite advancements in continuous glucose monitoring (CGM) technologies, insulin dosing remains predominantly reactive and static. These limitations underscore the need for intelligent, adaptive systems capable of personalized treatment.

With the rapid advancement of artificial intelligence (AI) and machine learning (ML) technologies, along with the growing availability of big data in health care, the integration of AI into health care is becoming increasingly feasible [5]. AI’s growing role in health care has driven innovations in areas such as image analysis, disease diagnosis and prognosis, clinical decision support, robotic surgery, virtual assistants, and drug target screening [6-9]. However, there are still challenges with the dynamic adaptiveness and explainability of the models. Thus, we leveraged the potential of AI and ML to develop an adaptive and explainable system for personalized insulin dosing for people with T1D.

Several studies [10-15] have examined ML methods for diagnosing and predicting the early onset of type 2 diabetes mellitus (T2DM). Most of these works have applied multiple ML algorithms such as decision trees, support vector machines, random forests, gradient boosting, k-nearest neighbors, neural networks, etc., using various demographics and clinical variables to diagnose and predict diabetes onset. These approaches often rely on static training paradigms and lack the adaptability needed to accommodate dynamic or nonstationary data distributions. Many studies [16-19] have explored ML approaches for BG prediction. These studies have used classical time series models, support vector machines, random forests, and long-short term memory models to predict the hypoglycemic events and forecast BG in near-time horizons. These studies are limited in their ability to provide personalized predictions due to an overreliance on CGM data, without incorporating additional contextual factors such as physical activity, carbohydrate intake, and insulin dosage. Furthermore, they often experience interpretability constraints.

The traditional models often lacked the adaptability required to respond dynamically to changes in a patient’s condition. This limitation paved the way for reinforcement learning (RL) models capable of continuous learning and adaptation. RL has attracted interest for its ability to dynamically optimize insulin dosing and predict glucose levels in real time by leveraging data from CGMs. There are some works focused on using RL for dynamic insulin recommendation and BG prediction [20-23]. Early works used Q-learning models [20], followed by more advanced methods such as deep Q-networks (DQNs) [21], actor-critic algorithms [23], and model-predictive control [22]. However, most of these models performed well in simulations; they required extensive manual tuning, making them impractical for long-term use in real-world settings. Thus, these approaches lack integration capabilities with real-world datasets or comprehensive evaluation against supervised models. Model explainability is another limitation in these RL-based methods, which is essential for clinical deployment. Additionally, previous studies frequently rely on synthetic datasets, offer limited explainability, and fail to benchmark RL against traditional machine learning models.

Despite the growing success of RL in health care applications, deploying these models in real-world clinical settings remains a challenge due to lack of interpretability in the models. Gottesman et al [24] discussed the practical hurdles of implementing RL models in health care, such as handling noisy data, model interpretability, and patient safety. Our work directly addresses these issues by applying Shapley additive explanations (SHAP) [25] and local interpretable model-agnostic explanations (LIME) [26] for better model explainability and by thoroughly testing the model on various patient datasets to ensure robustness and safety. Additionally, another study [27] aims to highlight the importance of simulating real-world conditions for RL models to ensure their generalization to unseen patient scenarios. We tackle this challenge by incorporating simulated testing and leveraging CGM data to train and test our RL model.

To address the above-mentioned limitations, we propose a DQN-based RL system that dynamically personalizes insulin dosing recommendations using CGM data, meal intake, and physical activity levels using a real-world dataset from the Ohio T1DM challenge [28], compare the effectiveness of the built model with benchmark ML models, and incorporate explainability using SHAP [25] and LIME [26].

Building on prior DQN-based insulin-dosing research, our paper emphasizes an explainability-first RL framework using the real-world multimodal OhioT1DM dataset. We integrate SHAP and LIME analyses with clinically relevant vignettes (eg, premeal bolus timing and postexercise hypoglycemia mitigation) to contextualize model behavior. Our safety-aware reward rationale is supported by a compact, systematic sensitivity design—including asymmetry, stability, and insulin on-board traces (IOB)-proxy components across multiple glycemic targets—and by a reproducible data pipeline that harmonizes pump or CGM data with wearable sensor signals. Finally, we implement leakage-safe data splits and report mean (SD) with 95% CIs for TIR or TBR or TAR and error metrics to enhance clinical interpretability and methodological rigor.

Our study uniquely focuses on model explainability—an essential factor for clinical trust that is largely overlooked in existing research. In this study, we applied SHAP to assess feature importance within the DQN model. SHAP values quantify the contribution of each input feature to the model’s decisions—for instance, illustrating how “time since last insulin dose” influences insulin recommendations. This explainability perspective is lacking in existing works, which have focused solely on the performance of RL algorithms without examining the rationale behind specific dosing decisions. Another distinguishing aspect of our work is the incorporation of LIME, which is used to explain individual insulin dose recommendations by approximating the DQN model with interpretable surrogate models. For instance, a clinician can interpret a recommended dose by analyzing contributing factors like meal timing or prior glucose patterns. By integrating SHAP and LIME, our work bridges the gap between black-box AI models and the level of interpretability necessary for regulatory approval and clinical implementation.

### Objective

We have developed a DQN-based RL system that dynamically personalizes insulin dosing recommendations using CGM data, meal intake, and physical activity levels, and has the capability to predict future BG levels at specified intervals (eg, 30 or 60 min) using historical event data. By leveraging real-time data, the model adapts to patients’ evolving physiological states, enhancing glucose control and patient safety. We have compared the effectiveness of our model with the benchmark ML models and incorporated explainability using SHAP and LIME to enhance model understanding. The model’s prediction can aid in forecasting episodes of hyperglycemia or hypoglycemia and inform optimal insulin dosing and lifestyle adjustments. Our major contributions in this study are as follows:

1. We have proposed and developed a novel and adaptive RL-based framework for personalized insulin dosing recommendations as well as predicting future BG levels. Our framework consists of three major components:
  - Deep Q-network: DQN uses a value-based approach where a neural network approximates the Q-value function to decide optimal actions for given states. This is distinct because DQN is better suited for discrete action spaces, which aligns well with insulin dosing (eg, no dose, low dose, medium dose, and high dose).
  - State space representation: this consists of lag features, rolling averages, and time-based features, offering a richer, temporally aware state representation. It explicitly models time since last insulin dose and time since last meal, emphasizing the physiological delay in glucose-insulin dynamics.
  - Reward design: the reward function penalizes extreme hypo- and hyperglycemia events (<70 and>250 mg/dL), with positive rewards for glucose levels within the target range (70‐180 mg/dL). Our approach introduces adaptive penalties, which vary with the severity of glucose deviation, potentially improving safety margins.
2. We have performed extensive evaluation of our RL-based framework performance with various metrics and benchmarked the performance with the state-of-the-art long short-term memory (LSTM) model.
3. We have incorporated explainability into the built RL-based model by integrating SHAP and LIME methods to assess feature importance and explain individual insulin dosing recommendations.

## Methods

### Ethical Considerations

The OhioT1DM dataset is a deidentified dataset requested from the authors through a data use agreement. No institutional review board review or approval is required because the data are completely deidentified.

### Dataset and Preprocessing

In this study, we used the OhioT1DM dataset from 2018 and 2020, provided by the Blood Glucose Level Prediction challenge [28,29]. The dataset was generated by monitoring 12 individuals with T1D over an 8-week period, capturing a range of BG-related data. It includes CGM readings recorded every five minutes, insulin delivery data from insulin pumps, and self-reported events such as meals, work, sleep, psychological stress, and physical activity, all logged via a smartphone app. Additionally, physical activity was tracked using a sensor band. The first cohort, consisting of 6 individuals, wore Basis Peak fitness bands; the dataset contains 5-minute aggregated measurements of heart rate, galvanic skin response (GSR), skin temperature, air temperature, and step count [28]. The second cohort, also comprising 6 individuals, wore the Empatica Embrace; the dataset provides 1-minute aggregated measurements of GSR, skin temperature, and acceleration magnitude [28]. Notably, meal and insulin data are represented as discrete user-entered values rather than continuous series like carbohydrate intake or insulin on board. The detailed data description is shown in Table 1.

**Table 1.** Dataset description.

Attributes	Description	Unit
timestamp	Date and time of the event	DateTime
glucose_level	Blood glucose level at a specific time	mg/dL
insulin_dose	Administered insulin dose during the event	Units (U)
carbs	Carbohydrate intake associated with a meal	grams (g)
meal_type	Type of meal (eg, breakfast, lunch, and snack)	—^a^
exercise_intensity	Intensity of physical activity	Intensity Level (1-5)
exercise_duration	Duration of the exercise	minutes
bolus_dose	Insulin dose delivered as a bolus	Units (U)
heart_rate	Heart rate during the event	beats per minute (bpm)
sleep_quality	Quality of sleep	Percent (%)

a Not applicable.

The OhioT1DM dataset consists of participant demographics: gender distribution (6 male, 6 female), age ranges (20‐80 y), and device use. All participants were on insulin pump therapy, using Medtronic 530G or 630G models with Medtronic Enlite CGM sensors. Both basal and bolus insulin data are included. While BMI and diabetes duration are not reported in the public dataset, all participants were experienced pump users, and therapy modalities were consistent across the cohort [28]. The detailed description is shown in Table S2 in Multimedia Appendix 1.

The raw dataset is in XML format. We converted the XML files into CSV format first, performed preprocessing steps such as handling missing data and normalizing features, and generated engineered features, including lag, rolling, and time-based attributes, for further analysis. We extracted relevant data such as glucose levels, insulin doses (from bolus events), meals (carbs intake), exercise, and other factors. The key characteristics of the extracted data are shown in Table 1. The extracted data consists of 1,191,753 records with the attributes as shown in Table 1.

The time is aligned to 5 minutes, and Empatica 1-minute channels are down-sampled (mean or median) to 5 minutes before fusion. Missing CGM gaps are imputed using backward or forward fill up to short horizon (<=15 min), and longer gaps are excluded and not being considered for learning or evaluation. The sensor outliers (eg, CGM<40 mg/dL or >400 mg/dL, nonphysiologic spikes) are clipped and flagged. The duplicate or overlapping basal and multipart bolus events (square or dual) are resolved to continuous IOB traces.

**Figure 1.**
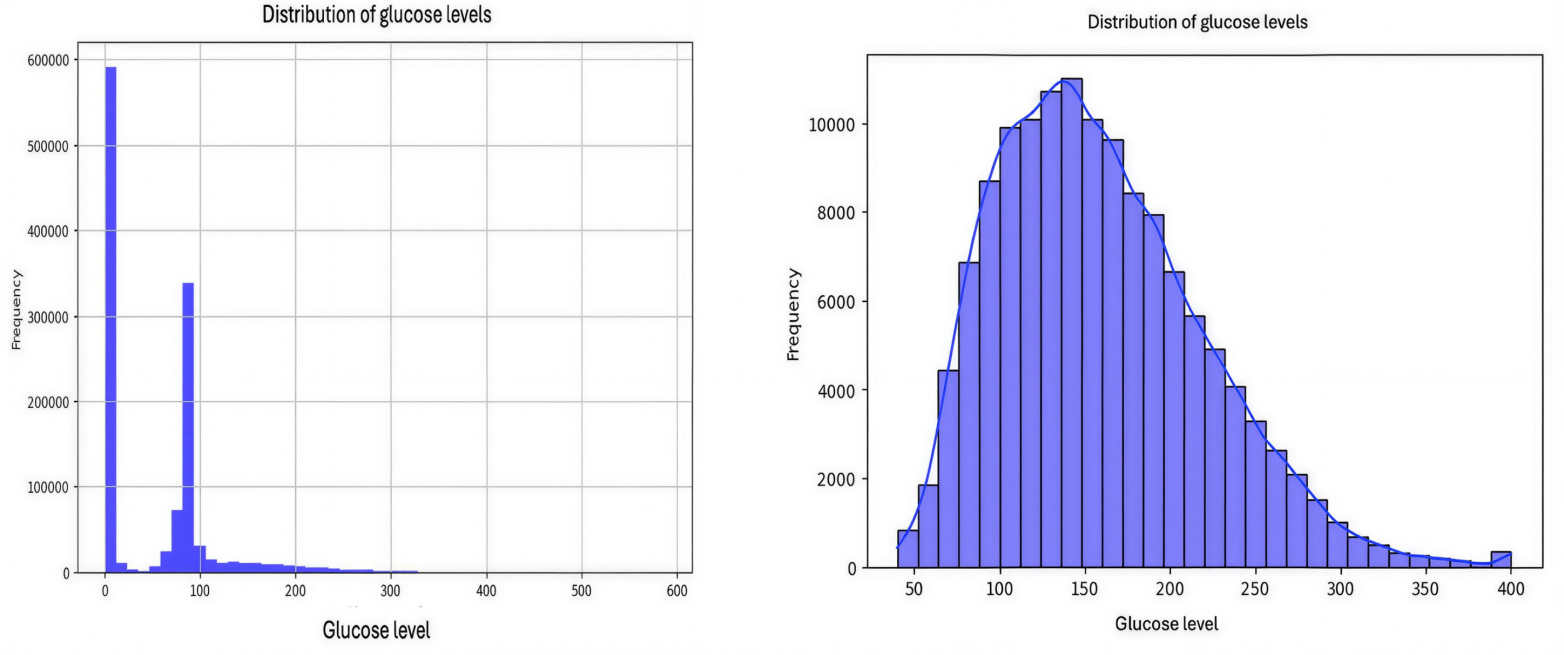
(A) Distribution of glucose levels (left) and (B) distribution of glucose levels in specific range (right).

Time series and distribution plots were used to identify trends, as illustrated in the figures. Figure 1A and B shows the distribution of glucose levels overall and within a specific range, respectively. As shown in Figure 1A, there is a highly skewed distribution of glucose levels where a significant number of entries have glucose levels close to 0, which could indicate periods of hypoglycemia or potentially erroneous readings. Figure 1B shows the distribution of glucose levels ranging from 50 to 400, with an average level of 150. There is another peak around 100, as shown in Figure 1B, which is closer to typical blood sugar levels but still slightly on the lower side of normal. There are fewer instances of high glucose levels, suggesting fewer episodes of high blood sugar in the dataset.

**Figure 2.**
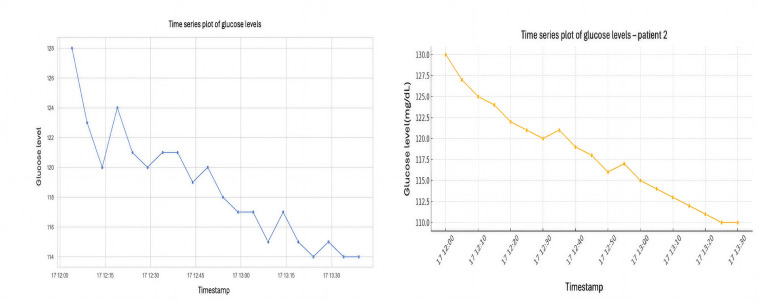
(A) Glucose levels variations for patient 1 (left) and (B) glucose levels variations for patient 2 (right).

Figure 2A and B illustrate the glucose levels over time for 2 sample patients from the dataset. Given that glucose levels are timestamped, we analyze how these levels change over time within individual patients and across different days or times of day. We can observe a general downward trend, with minor fluctuations. This suggests the potential usefulness of considering past values (lag features) when predicting future glucose levels. The descriptive statistics for all 12 participants’ data are in Table S1 in Multimedia Appendix 1.

### Model Development

RL [30] focuses on the task of deriving a policy that maps states to actions in a way that maximizes cumulative reward. These problems are inherently closed-loop, as the agent’s actions directly affect subsequent environmental states and observations. Unlike in supervised learning, the agent receives no explicit instruction on which actions to take; it must learn the optimal actions through interaction with the environment, identifying those that maximize reward via trial and error. RL has been successfully applied across various scientific domains, including robotics and control systems [31], manufacturing, and combinatorial search tasks like those found in computer games [32,33]. In the health care domain, RL has leveraged historical medical data, such as medical images and treatment regimens, for tasks including cancer prediction, diagnosis, and prognosis [24,34,35].

RL systems have multiple components: an agent, a policy, a reward signal, a value function, and optionally a model for the environment [30]. In RL, the goal is for an agent to learn a policy that maximizes a cumulative reward through interacting with an environment. A policy represents a mapping from states to actions that dictates the agent’s behavior at a specific point in time. It aligns with the concept of stimulus–response associations in psychology, where the term ’stimulus’ encompasses both external inputs and internally generated cues within the organism [30]. A reward signal defines the goal of a problem. At each time step, the environment provides the RL agent with a numerical reward. The agent’s primary objective is to maximize the cumulative reward it obtains over time [30]. While the reward signal reflects the immediate desirability of a given outcome, the value function captures long-term benefit. Specifically, the value of a state represents the expected cumulative reward an agent can obtain in the future, beginning from that state [30]. The fourth and final component present in some RL systems is a model of the environment. This model simulates the environment’s dynamics or, more broadly, enables the agent to make predictions about future environmental responses [30].

We trained a DQN agent to maximize time in target glucose range. States included glucose, insulin, meals, and exercise. Actions were discrete insulin doses. Reward function penalized values outside 70‐180 mg/dL. The core RL equation is the Bellman equation, which forms the basis for algorithms such as Q-learning and DQN.

The Bellman Equation for Q-Learning:


(1) Q(s,a)=r+γmaxa′Q(s′,a′)

- Q (s, a) is the Q-value (the expected future reward) for taking action *a* in state *s*.
- r is the immediate reward received after taking action *a* in state *s*.
- γ is the discount factor (typically between 0 and 1), which determines how much future rewards are worth compared to immediate rewards.
- maxa′Q (s′, a′) is the maximum future Q-value (the best future reward the agent can achieve) for the next state *s′* and all possible actions *a′*.

The Bellman equation details with respect to the problem are described below:

1. State (s): in the context of diabetes management, the state can be represented as a combination of features such as the current glucose level, insulin dose, meal intake, exercise, etc
2. Action (a): the action refers to the insulin dose recommendation or adjustment (eg, deciding how much insulin to administer at the current state).
3. Reward (r): the reward is a feedback signal to indicate the success of the agent’s action. In diabetes management, the reward might penalize for hypo- or hyperglycemia events and give positive rewards for keeping glucose levels within a healthy range.
4. Discount factor (γ): the discount factor controls the balance between prioritizing immediate rewards (eg, maintaining glucose levels right now) and future rewards (eg, preventing long-term health issues caused by poor glucose control).
5. Max future Q-value: the agent estimates the maximum future reward it can achieve by taking the best action in the next state. This is used to help the agent choose actions that will not only lead to immediate benefits but also to longer-term gains.

### Reinforcement Learning Model Selection

The following steps describe the reinforcement learning model selection and training process:

1. Build and train a reinforcement learning model: develop a DQN model to optimize insulin dosing based on extracted parameters such as glucose levels. Details for the DQN model are in the section below.
2. Evaluate and optimize the RL model: after training, we evaluate the RL model’s performance using both datasets [28,29], optimize it, and detail the evaluation metrics.
3. Establish state representation: BG levels, insulin doses, meal intake, and physical activity over a recent time window (eg, 2 h).
· Reward function: a penalized reward system that reduces points for glucose levels outside the target range, with larger penalties for extreme hypo- or hyperglycemia events.
5. Implement the training loop for the DQN agent to interact with our diabetes management environment by setting up the environment and the agent, then running through multiple episodes to allow the agent to learn optimal actions based on the given state.
6. We set up the DQN agent and its learning mechanisms, and we train this agent using the simulation environment by repeatedly interacting with it (using the step function) and applying the replay function to learn from past actions.

### DQN Rule

In the context of DQNs, the Bellman equation is approximated using neural networks to learn the Q-value function. The update rule is:


(2) Q(s,a)←Q(s,a)+α(r+γmaxQ(s′,a′)−Q(s,a))a′

- *α* is the learning rate that controls how much the Q-values are updated at each step.
- The expression *r+γmaxa′Q(s′,a′*) is the target Q-value, and the difference *r+γmaxa′Q(s′,a′)−Q(s,a*) is the temporal difference error.

### Details With Respect to the Problem

The following key concepts are central to understanding the problem formulation in reinforcement learning–based diabetes management systems:

1. Policy: the policy defines the agent’s behavior — which actions to take in different states. The agent seeks to learn an optimal policy that maximizes the total reward.
2. Exploration versus exploitation: to learn the best policy, the agent must explore different actions (exploration) while also choosing actions it believes will give the best reward based on its current knowledge (exploitation).

In the diabetes management system, the agent continuously learns to recommend insulin doses based on real-time data such as glucose levels, insulin history, and meal intake, balancing immediate glucose control with long-term health management.

### Feature Engineering

We have extracted below features after data analysis:

1. Lag features: these capture the prior values of glucose levels. Given the data collection frequency of every 5 minutes, we have created lag features representing glucose levels from 30 to 60 minutes prior.
2. Rolling window features: these features consist of rolling averages to smooth out fluctuations and capture trends over time, containing rolling means and standard deviations over various windows, such as 30 minutes and 60 minutes.
3. Time-based features: since physiological responses can vary throughout the day, including features such as the hour of the day, might capture these variations effectively. We extracted time-based features from the timestamp such as the hour of the day.

### Evaluation Metrics

The performance was evaluated quantitatively using standard metrics such as mean absolute error (MAE), root mean square error (RMSE), and time-in-range (TIR). MAE [36] and RMSE [37] are used to assess the accuracy of predictive models for glucose forecasting; however, TIR [38] represents the percentage of time a person’s glucose levels remain within the target range, typically 70‐180 mg/dL for most adults with diabetes.

The MAE [36] is the average of the absolute errors (ie, the difference between the actual and predicted glucose values).


(3) $$MAE=\frac{1}{n}\sum_{i=1}^{n}\left|y_{predictedi-}y_{actuali}\right|$$

It can be represented, as shown in Equation 3, where n is the number of test instances. A lower MAE value leads to a better model.

RMSE [37] is the square root of the average of squared errors (ie, the difference between the actual and predicted popularity values). RMSE can be represented as shown in Equation 4, where n is the number of test instances. A lower RMSE value leads to a better model.


(4) $$RMSE=\;\sqrt{\frac{1}{n\;}\;\sum_{i=1}^{n}{\left(y_{predictedi}-\;y_{actuali\;}\right)}^{2}}$$

TIR is represented in Equation 5, where higher TIR is associated with better glucose control as compared to lower TIR values.


(5) $$TIR=\left(\frac{Total\;count\;of\;glucose\;reading\;within\;range}{Total\;number\;of\;reading}\right)\times 100$$

### Hypothesis

We expect the RL-based model to outperform baseline approaches by increasing the percentage of time glucose levels remain in the target range while minimizing hypo- and hyperglycemia events.

### Simulation Outcome

#### State

The following describes the simulation state:

- Glucose level: 80.06 mg/dL, which falls within the target range (70‐180 mg/dL). This is a healthy and realistic level for a patient managing diabetes, indicating that the simulation now better reflects the models’ physiological responses to insulin.
- Time since last meal: 1 hour, which is a typical scenario and can influence immediate subsequent readings.
- Time since last dose: 1 hour, reflecting recent insulin activity, which might be stabilizing BG.
- Insulin type: generic bolus (mealtime) and basal (background) insulin doses.
- Time of day: 12 (noon), a common time for a meal which might coincide with a postprandial glucose reading.

#### Reward

The maximum positive reward is 10, reflecting optimal glucose management in the simulation. This reward confirms that the glucose level is within the desired range, and the model’s reward system is functioning as intended to encourage similar outcomes. The adjusted parameters helped align the simulation more closely with realistic diabetes management scenarios. The positive reward outcome encourages the model to replicate or aim for similar decisions under comparable circumstances, reinforcing good management practices. We implemented a conservative, interpretable reward function prioritizing glycemic safety, consisting of in-range bonuses (70‐180 mg/dL) and out-of-range penalties, with an asymmetric variant that assigns stronger penalties to hypoglycemia than to hyperglycemia. To evaluate robustness, we conducted a structured sensitivity analysis across (1) alternative glycemic thresholds (70‐180, 80‐160, and 70‐140 mg/dL) and (2) incremental reward components, including the baseline formulation, asymmetric penalties, a stability penalty on |ΔG/Δt| (ie, penalizes rapid glucose changes reducing glycemic variability where ΔG represents the glucose change with respect to time t), and an IOB proxy penalty to discourage insulin stacking. Across conditions, we summarize TIR or TBR or TAR and MAE or RMSE, showing that safety-focused asymmetry consistently reduces TBR with minimal impact on TIR. These findings support the use of a simple yet clinically aligned reward structure for this initial study.

## Results

### Experimental Setup

The models were trained for 10 episodes using simulated environments initialized with realistic patient data extracted from the OhioT1DM dataset. The model architecture consisted of a 3-layer fully connected neural network with ReLU activations, Adam optimizer (learning rate 0.001), epsilon-greedy exploration (ε decay from 1.0 to 0.1 over episodes), and a replay buffer size of 5000. We have used time-blocked split per subject consisting of weeks 1‐6 as the training set, week 7 as validation, and week 8 as the test set. We have used TensorFlow 2.12 for neural network implementation of DQN agent, OpenAI Gym for simulating the agent’s interaction with glucose trajectories and insulin dosing responses, Pandas and NumPy for data preprocessing and state-space formulation, and Matplotlib and seaborn for data visualization. The environment represented each state as a vector composed of time-series values over a 2-hour sliding window of glucose levels, meal carbohydrate intake, physical activity intensity or duration, and prior insulin doses. The experiments were conducted on a local machine with Apple M1 Pro 10-core CPU and 32 GB RAM. Training durations were intentionally limited to 10 episodes due to computational constraints, which balances exploratory learning without excessive runtime. The learning curves showed stabilization by episode 8, with negligible performance improvement thereafter (ΔTIR<0.5%). The learning curves demonstrated an early plateau in episodic reward and TIR, indicating initial stabilization but not the full convergence typically expected in deep RL. Future work will extend training to at least 300 episodes, incorporate soft target updates (τ=.005), maintain a replay buffer of at least 100 k transitions, decay exploration to *ε*=.05, and use leave-one-subject-out (LOSO) cross-validation. Each episode consisted of ~1000‐2000 state transitions (steps), depending on the patient’s data length and time granularity. To ensure reproducibility, random seeds were set for NumPy and TensorFlow. Model checkpoints were stored and reloaded for inference and evaluation. Performance metrics such as MAE, RMSE, and TIR% were computed on unseen test data segmented from each patient’s profile. Reward shaping and epsilon decay were used for policy exploration. Our study provides valuable insights into the performance of the DQN agent in managing glucose levels through insulin dosing.

### Integration With DQN Algorithm for Training

The simulation environment produces realistic and consistent results when we integrate with the DQN model for training. We initialize the DQN Agent by setting up the neural network model that will learn the Q-values. Then simulate interactions while running episodes where the agent interacts with the environment, makes decisions based on its current policy, observes rewards, and updates its policy accordingly. We then proceeded with the training loop where for each episode, reset the environment. For each time step within the episode, choose an action from the DQN agent, observe the new state and reward, and store this experience. And periodically update the DQN agent’s neural network by replaying a batch of experiences.

### Model Performance

We compared the glucose prediction performances of two models (ie, DQN and LSTM) in this study. We observed that DQN achieves a slightly lower RMSE (12.39) as compared to LSTM (12.87), indicating it makes fewer large-scale prediction errors. RMSE penalizes larger errors more heavily, so this suggests that DQN is better at avoiding extreme outliers in glucose level prediction. Moreover, DQN is more stable and robust in maintaining glucose predictions closer to the true values, particularly in high-variance regions. LSTM achieves a significantly lower MAE (3.69), suggesting its day-to-day average predictions are closer to the actual glucose levels than DQN’s. However, MAE does not penalize outliers as strongly as RMSE. So, while LSTM is generally more accurate on average, it is more vulnerable to large mistakes, as reflected by its higher RMSE.

**Figure 3.**
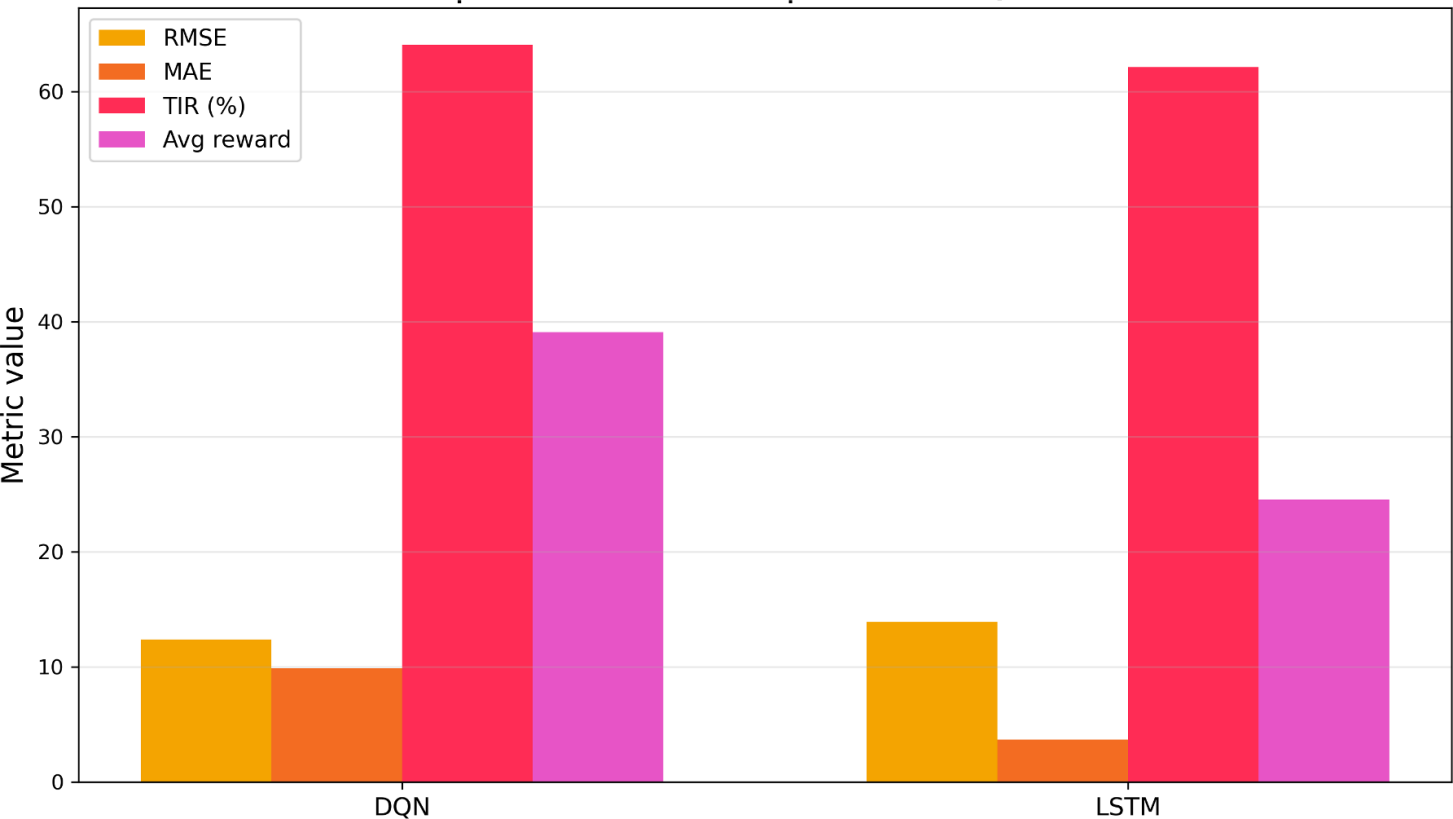
Model performance comparison for deep-Q-network with LSTM. DQN: deep-Q-network; LSTM: long short-term memory; MAE: mean absolute error; RMSE: root mean square error; TIR: time-in range.

The DQN model (64.06%) outperforms LSTM (62.10%) slightly for TIR. This is critical in real-world diabetes management, where maintaining glucose in a safe range directly correlates with reduced risk of complications. Since DQN is an RL-based model, reward is a key internal measure of how well it is optimizing glucose control based on its policy. A higher average reward for DQN (39.09) indicates it is more effective in learning strategies that lead to favorable outcomes (ie, TIR maintenance and avoiding hypo- or hyperglycemia). LSTM is not a reinforcement model, so its reward is derived post hoc and may not reflect learning behavior but rather performance fitting (Figure 3).

Table 2 summarizes the comparative insights for the model performance using all evaluation metrics between DQN and LSTM models.

**Table 2.** Summary of comparative insights.

Dimension	Better model	Reason
RMSE^a^	DQN^b^	Fewer large-scale prediction errors
MAE^c^	LSTM^d^	Closer average prediction to ground truth
TIR^e^ (%)	DQN	Maintains glucose in safe range longer
Reward	DQN	Optimizes policy to reinforce good outcomes

a RMSE: root mean square error.

b DQN: deep-Q-network.

c MAE: mean absolute error.

d LSTM: long short-term memory.

e TIR: time in range.

Table 3 summarizes the statistical analysis of model performance between DQN and LSTM models. On average, the RMSE across models is about 13.1 mg/dL. The relatively small SD shows little variability between DQN (12.39) and LSTM (13.87). The wide CI reflects the small sample size (only 2 models), so additional baselines would stabilize the estimate. The MAE varied more strongly between models (DQN=9.85 vs LSTM=3.69), leading to a large SD and an unrealistic CI range. This shows that comparing only 2 models provides limited inferential strength. LSTM had notably lower MAE, which suggests better average prediction closeness, though this must be contextualized with other metrics. Both models kept patients in the clinical target range (70‐180 mg/dL)~63% of the time. The small SD indicates stable performance between DQN (64.06%) and LSTM (62.10%). Still, the CI is wide because of limited sample points; more repeated trials are needed to confirm robustness. The reward variance is high due to differences in RL design. DQN achieved a much higher average reward (39.09) than LSTM (24.54). The wide CI reflects the volatility of the RL process and the insufficient number of episodes (only 10, per earlier feedback).

**Table 3.** Summary statistics of model performance.

Metric	Mean (SD)	95% CI
RMSE^a^	13.13 (1.05)	(3.73-22.53)
MAE^b^	6.77 (4.36)	(–32.37 to 45.91)
TIR^c^ (%)	63.08 (1.39)	(50.63%-75.53%)
Reward	31.82 (10.29)	(–60.62 to 124.25)

a RMSE: root mean square error.

b MAE: mean absolute error.

c TIR: time in range.

Overall, RMSE and TIR are stable across models, suggesting both frameworks maintain reasonable glucose control. The MAE favors LSTM, but this may be influenced by LSTM’s smoother short-term predictions, whereas DQN optimizes long-term control. The reward strongly favors DQN, aligning with its reinforcement design, but instability remains due to a few training episodes. Statistical confidence is weak due to a very small sample size (n=2 models); adding baselines (eg, autoregressive integrated moving average, random forest, and simple linear predictors) would allow meaningful variance testing (ANOVA).

### Explainability Analysis of RL Model

The RL model provides a clinically aligned strategy for insulin management. It not only predicts future glucose values but acts upon them in a self-improving feedback loop, a defining characteristic of RL. We implemented an explainability approach in the model using techniques such as SHAP and LIME, so that clinicians can understand these decisions, aiding interpretability and safety validation in future real-world trials.

### SHAP Summary Plot

**Figure 4.**
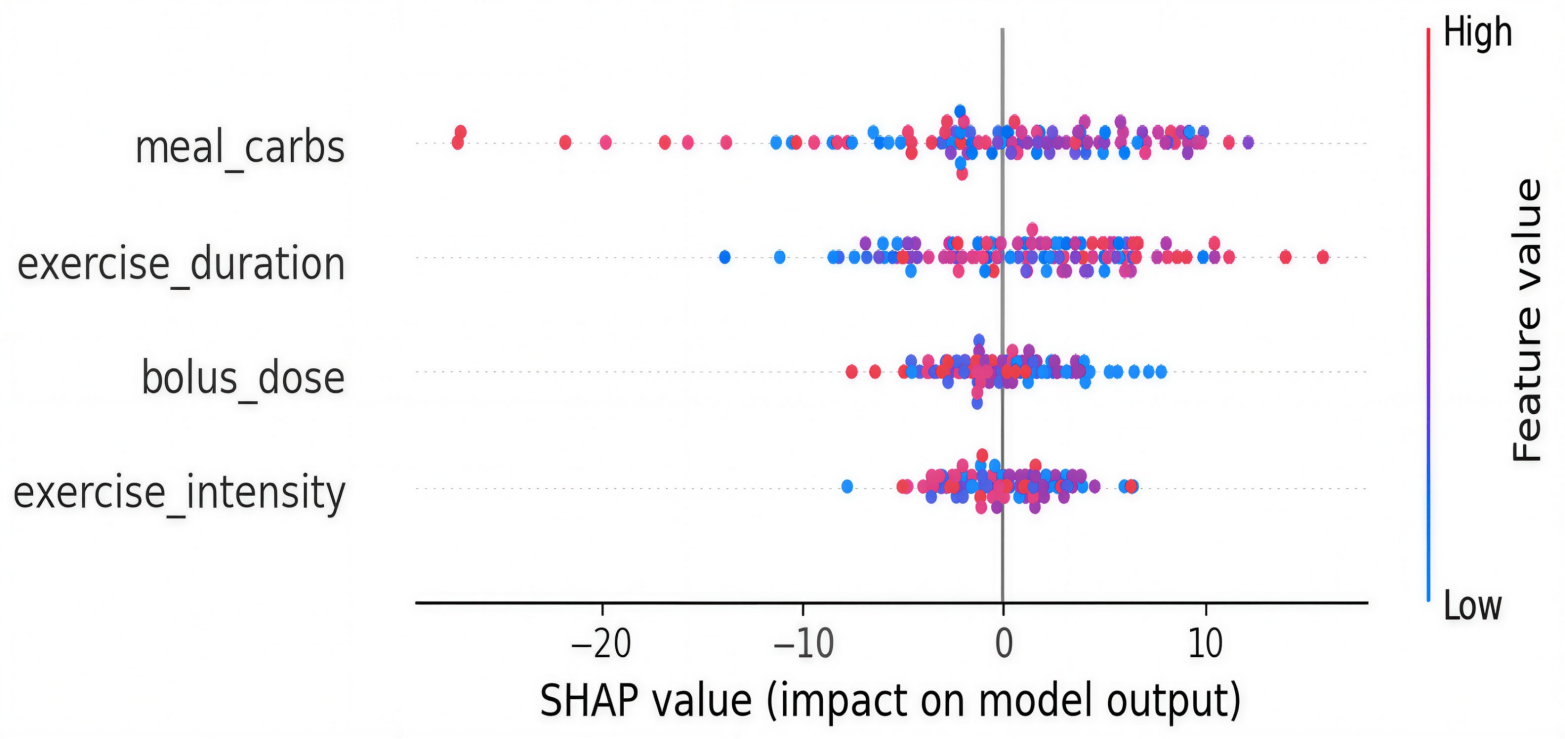
SHAP plot. SHAP: Shapley additive explanations.

The SHAP [25] summary plot provides a global view of the feature importance and direction of impact on the model’s glucose level predictions. We have applied the SHAP method to improve the interpretability of our RL model. The feature values are represented in color from low (blue) to high (red), and their impact (SHAP value) is plotted on the x-axis as shown in Figure 4.

As shown in Figure 4, “meal_carbs” shows the highest SHAP impact range, varying from approximately −25 to +10. High meal_carbs (red) significantly increase predicted glucose. “exercise_duration” ranges in SHAP values from about −10 to +10, indicating that longer durations can both decrease or modestly increase glucose depending on context. *“*bolus_dose” mostly impacts glucose predictions negatively, ranging from −10 to +5 SHAP units, where higher doses (red) tend to lower glucose predictions. *“*exercise_intensity” exhibits mostly negative SHAP values clustered between −7 and 0, reflecting a minor downward pressure on glucose levels with increased intensity.

### SHAP Dependence and Interaction Effect of Meal Carbs Feature With Exercise Duration

**Figure 5.**
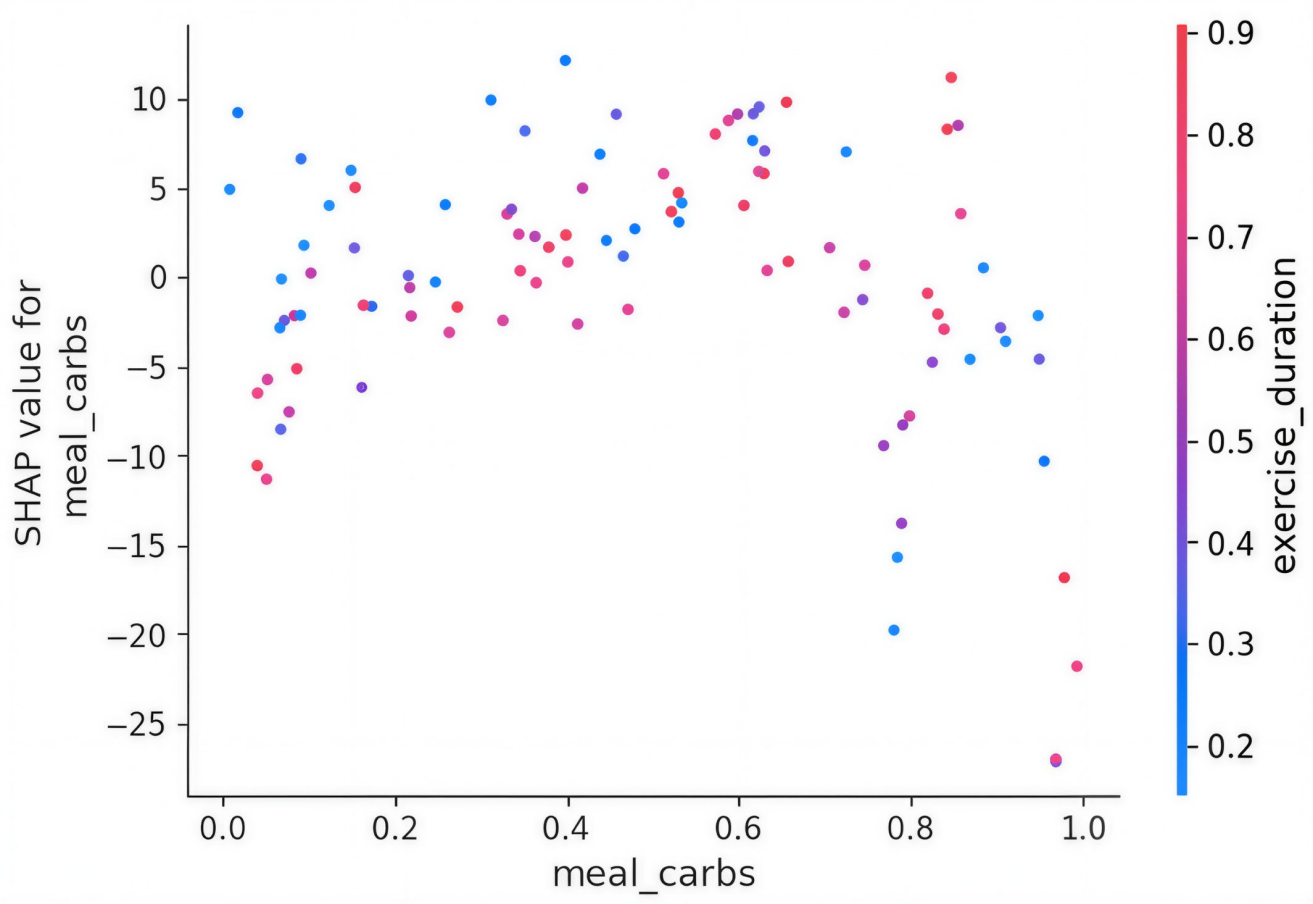
SHAP interaction plot. SHAP: Shapley additive explanations.

Figure 5 shows the interaction plot of features meal carbs with exercise duration and their impact on the SHAP values. This dependence plot explores the impact of “meal_carbs” on the SHAP values, which reflects its influence on glucose predictions. The plot also colors each point by “exercise_duration” to show interaction effects, as shown in Figure 5.

When *“*meal_carbs*”* is normalized below 0.2, SHAP values mostly fall between −5 and +5, showing minimal impact. As *“*meal_carbs” increases to the range of 0.4‐0.6, SHAP values often rise above +5, with some reaching ~+10. At very high “meal_carbs” values (near 1.0), SHAP values can reach as low as −25 and as high as +10. Points with lower *“*exercise_duration” (more pink or red) cluster with higher positive SHAP values, indicating increased glucose risk when carbs are high and exercise is low.

### LIME Explanation Plot for Individual Predictions

**Figure 6.**
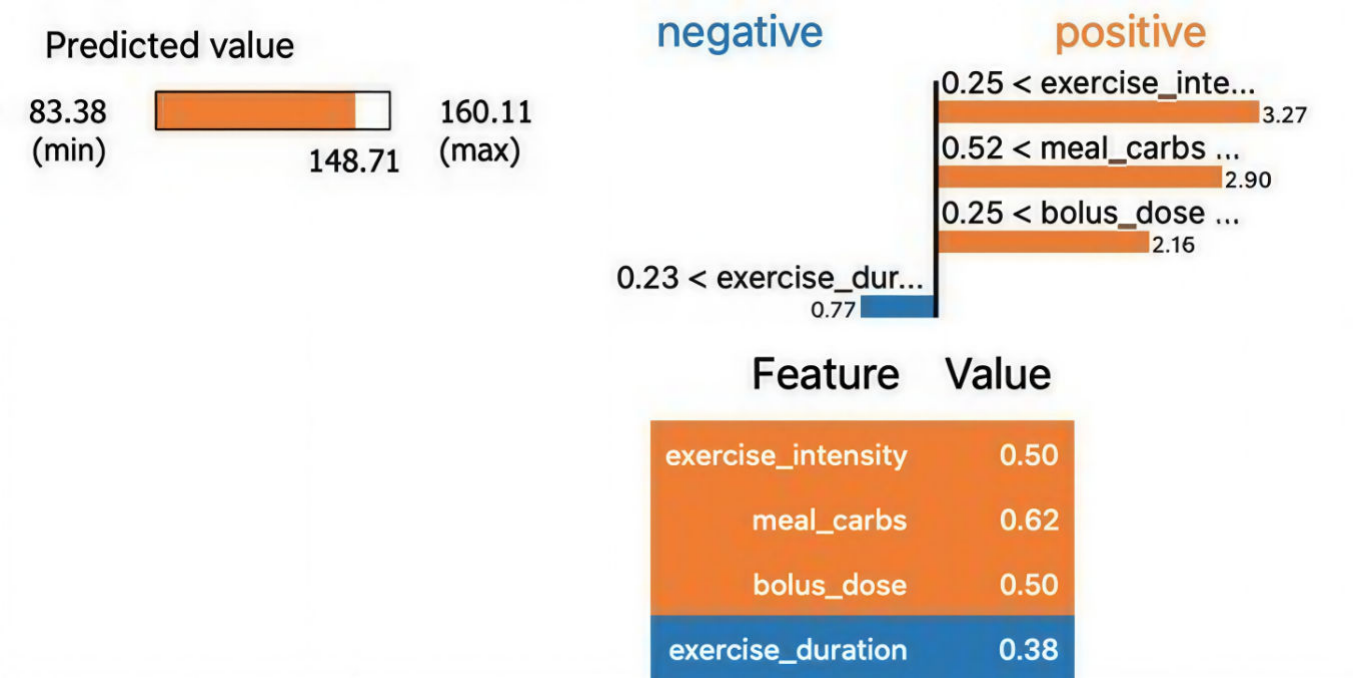
Local interpretable model-agnostic explanations plot.

The LIME plot presents the local feature contribution to a single glucose level prediction made by the model. Figure 6 shows the LIME plot to explain the individual features’ contribution in the glucose prediction. The positive and negative bars show how much each feature pushed the predicted value up or down for this instance.

As shown in Figure 6, we observed predicted glucose value: ~148.71 mg/dL (within the model’s learned bounds of 83.38 to 160.11 mg/dL). The *“*meal_carbs” feature at 0.62 contributed +8.27 units to glucose prediction while *“bolus_dose*” at 0.50 contributed +2.90 units. This might indicate a mismatch or delay in insulin effect. The *“*exercise_intensity” feature at 0.50 contributed +2.27 units while “exercise_duration*”* feature at 0.38 contributed −0.77 units, helping to modestly reduce predicted glucose.

### Clinical Interpretation and Implications

Our model explainability results uncover how key variables influence BG predictions by combining global (SHAP) and local (LIME) interpretability methods. The key takeaways include: high meal carbohydrate intake is a dominant factor raising glucose, especially when not balanced by insulin or physical activity. Insulin dosing (*“*bolus_dose”) generally reduces glucose but can be insufficient if the meal size is high or if timing is off. Exercise, particularly duration, helps buffer glucose spikes and improves prediction outcomes. Personalized decision-making should consider the interaction between carbs, insulin, and exercise—simple rule-based systems may overlook these subtleties. This reinforces the need for dynamic, explainable AI systems in diabetes management to tailor recommendations based on a full patient context.

We ground the interpretability in patient-facing and clinician-facing scenarios, using concrete values we already computed: For example (LIME, single decision): predicted glucose=148.7 mg/dL; features contributing upward: “meal_carbs=0.62” (+8.27 SHAP or LIME units), “bolus:_dose=0.50” (+2.90 units; likely timing mismatch vs IOB), “exercise_intensity=0.50” (+2.27 units), and “exercise_duration=0.38” (−0.77 units) lowering risk. The clinical actionability example is:

1. Premeal guidance: if SHAP shows carbs consistently the top driver (+6‐10 units) and low IOB, suggest pre-bolus timing (eg, 15‐20 min) or carb ratio adjustment for that period of day.
2. Postexercise hypoglycemia risk: if SHAP highlights high exercise_duration with negative contributions and declining rate-of-change, the system can warn: reduce correction bolus or add carbs to avoid late-onset lows.
3. Overnight stability: if basal-driven negative SHAP at night with frequent lows, suggest basal down-titration or tighter overnight safety constraints in the policy.

## Discussion

### Principal Findings

In summary, our results suggest that DQN outperformed static dosing models and matched supervised models such as LSTM in RMSE while offering dynamic control. Explainability analysis revealed critical decision factors. An RMSE of 12.39 mg/dL is also fairly low but slightly higher than the MAE, suggesting that there might be occasional predictions with larger errors. Despite this, the model’s overall predictive accuracy is good. Percentage time in target range (64.06%). This metric indicates that the glucose levels are within the clinically safe range (70‐180 mg/dL) about 64.06% of the time. Although this represents the majority of the time, there remains room for improvement, as optimal diabetes management typically aims for a higher percentage within this range to minimize the risk of complications from high or low BG levels. An average reward of 39.09 indicates that the agent generally performs well under the reward structure we have defined, which presumably rewards the agent for maintaining glucose levels within the target range and penalizes it for deviations.

### Clinical Relevance

Explainable RL can improve clinician trust, facilitate regulatory approval, and ensure patient safety. Adaptive insulin recommendations reduce the risk of adverse glycemic events. General observations show that the majority of values lie below 200 mg/dL, which is generally considered within a manageable range for people with diabetes but includes many readings below 70 mg/dL, which are hypoglycemic. The upward trend in the data visualization suggests an increase in average glucose levels over time, though this could also reflect variabilities in patient behavior or treatment efficacy. The output from the simulation showed a glucose level of approximately 80.06 mg/dL and a reward of 10, suggesting that the adjustments to the insulin sensitivity and decay parameters have significantly improved the realism and functionality of the environment. DQN is superior in handling real-time decisions and dynamic conditions, making it more suitable for adaptive insulin dosing in personalized care. LSTM could be valuable in applications where predicting general trends is sufficient (eg, retrospective analysis and forecast dashboards). Integrating hybrid architectures (eg, DQN for policy and LSTM for predictive enhancement) may yield optimal results. Ultimately, DQN’s better TIR and reward profile signal its strength in closed-loop, autonomous glucose management systems.

### Limitations and Future Work

Although the results are promising, our work has certain limitations. First, the data were limited to 12 participants which limits the applicability of our work to real-time deployment. However, it provides enough evidence of using RL-based models for glucose prediction and insulin dosing recommendations. A larger dataset will help to validate the models better. The future work includes hybrid DQN-LSTM ensembles, generalization to Type 2 diabetes. We aim to apply continuous enhancement of the models to optimize performance by focusing on key components such as:

1. Data quality and quantity: ensuring high-quality and comprehensive data can help improve model accuracy. This includes a detailed recording of insulin doses, meals, exercise, glucose levels, etc.
2. Feature engineering: explore different features or combinations that might improve model predictions, such as time of day, preceding meal types, or exercise intensity.
3. Model tuning: for LSTM and DQN, parameter tuning could optimize performance. This involves adjusting learning rate, number of trees, depth of trees, etc.
4. Ensemble techniques: combining predictions from different models (eg, an ensemble of LSTM and DQN) might leverage strengths and mitigate individual weaknesses.
5. Incremental training: for LSTM and DQN, consider using an incremental training approach to continually update the models as new data becomes available, which might help in adapting to changes in patients’ lifestyle or insulin sensitivity.

### Conclusions

We demonstrate that a DQN-based RL system can effectively personalize insulin dosing in T1D. The system achieved strong predictive performance, maintained glucose within safe ranges, and enhanced interpretability through SHAP and LIME. This work paves the way for clinically integrated, AI-assisted diabetes care systems. The analysis shows that while the insulin type does not lead to significant differences in glucose control, individual management strategies, including timing and dosage adjustments, are critical. The variability seen both within and across patients suggests the need for personalized diabetes management plans, closely monitored by health care providers. The frequent fluctuations and the presence of high and low extremes indicate a need for a reinforcement learning model that can dynamically adjust insulin dosages based on CGM data to better manage and stabilize glucose levels. In reinforcement learning models such as DQN, the reward function plays a key role. If the reward function is not designed correctly, the model might not learn the correct associations between actions and outcomes (predictions). A poor reward signal could result in the model always predicting values close to zero.

## Supplementary material

10.2196/79195 Multimedia Appendix 1 Descriptive statistics of glucose levels and cohort characteristics.

## Abbreviations

AI: artificial intelligence
CGM: continuous glucose monitoring
DQN: deep Q-network
IOB: insulin on-board
LIME: local interpretable model-agnostic explanations
LR: logistic regression
LSTM: long short-term memory
MAE: mean absolute error
RL: reinforcement learning
RMSE: root mean square error
SHAP: Shapley additive explanations
T1D: type-1 diabetes
TIR: time in range
