# Supplementary material for: Personalized Type 1 Diabetes Management: Reinforcement Learning–Based Insulin Dosing and Glucose Forecasting
Source: JMIR Diabetes. 2026 Jun 3;11:e79195. doi: 10.2196/79195 (PMC13233011; doi:10.2196/79195)
Supplement: Multimedia Appendix 1 [file diabetes-v11-e79195-s001.docx]

**Supplementary Material**

**Table S1: Descriptive Statistics Summary of blood glucose level for N=12 participants**

| **Participant id** | **Counts** | **mean** | **std** | **min** | **25%** | **50%** | **75%** | **max** |
| --- | --- | --- | --- | --- | --- | --- | --- | --- |
| 1 | 96969 | 43.43 | 55.99 | 0.01 | 0.53 | 1.07 | 87.80 | 369 |
| 2 | 169831 | 38.80 | 53.57 | 0.01 | 0.98 | 1.05 | 87.24 | 400 |
| 3 | 86047 | 41.09 | 55.21 | 0.00 | 0.37 | 1.09 | 83.99 | 361 |
| 4 | 61279 | 78.22 | 61.63 | 0.00 | 56.00 | 82.94 | 90.50 | 586 |
| 5 | 63954 | 79.71 | 54.145 | 0.00 | 68.00 | 86.54 | 97.00 | 465 |
| 6 | 128002 | 41.28 | 56.21 | 0.01 | 0.90 | 1.06 | 88.63 | 462 |
| 7 | 63293 | 82.96 | 65.09 | 0.00 | 66.00 | 83.30 | 90.32 | 377 |
| 8 | 65765 | 74.45 | 54.09 | 0.00 | 46.00 | 83.30 | 89.60 | 428 |
| 9 | 153260 | 42.50 | 61.02 | 0.00 | 0.92 | 1.26 | 84.49 | 400 |
| 10 | 67791 | 81.31 | 58.34 | 0.00 | 60.00 | 86.36 | 94.10 | 413 |
| 11 | 63363 | 74.97 | 55.98 | 0.00 | 50.00 | 82.58 | 89.60 | 422 |
| 12 | 114131 | 40.92 | 54.35 | 0.01 | 0.08 | 1.06 | 87.33 | 367 |

**Table S2: Cohort description and clinical characteristics**

| **ID** | **Gender** | **Age** | **Pump Model** | **Sensor Band** | **Cohort** |
| --- | --- | --- | --- | --- | --- |
| 540 | male | 20-40 | 630G | Empatica | 2020 |
| 544 | male | 40-60 | 530G | Empatica | 2020 |
| 552 | male | 20-40 | 630G | Empatica | 2020 |
| 567 | female | 20-40 | 630G | Empatica | 2020 |
| 584 | male | 40-60 | 530G | Empatica | 2020 |
| 596 | male | 60-80 | 530G | Empatica | 2020 |
| 559 | female | 40-60 | 530G | Basis | 2018 |
| 563 | male | 40-60 | 530G | Basis | 2018 |
| 570 | male | 40-60 | 530G | Basis | 2018 |
| 575 | female | 40-60 | 530G | Basis | 2018 |
| 588 | female | 40-60 | 530G | Basis | 2018 |
| 591 | female | 40-60 | 530G | Basis | 2018 |
